# Conventional herniorrhaphy followed by laparoscopic appendectomy for a variant of Amyand’s hernia: a case report

**DOI:** 10.1186/s13256-023-04340-y

**Published:** 2024-03-30

**Authors:** Yau-Ren Chang, Yu-Tung Wu, Chi-Hsun Hsieh

**Affiliations:** Division of Trauma and Emergency Surgery, Department of Surgery, Linkou Chang Gung Memorial Hospital, Taoyuan, Taiwan

**Keywords:** Amyand’s hernia, Ruptured appendicitis, Incarcerated inguinal hernia

## Abstract

**Background:**

Amyand’s hernia (AH) is an appendix (with or without acute inflammation) trapped within an inguinal hernia. Most AH with acute appendicitis had a preexisting appendix within the hernia sac. We herein report a variant of AH that has never been described before. An inflamed appendix that was managed conservatively was found to have migrated and trapped in the sac of a previously unrecognized right inguinal hernia 6 weeks after the index admission, resulting in a secondary Amyand’s hernia.

**Case presentation:**

A 25-year-old healthy Taiwanese woman had persistent right lower abdominal pain for 1 week and was diagnosed with perforated appendicitis with a localized abscess by abdominal computed tomography (CT). No inguinal hernia was noted at that time. Although the inflamed appendix along with the abscess was deeply surrounded by bowel loops so that percutaneous drainage was not feasible, it was treated successfully with antibiotics. However, she was rehospitalized 6 weeks later for having a painful right inguinal bulging mass for a week. Abdominal CT revealed an inflamed appendix with abscess formation in an indirect inguinal hernia raising the question of a Amyand’s hernia with a perforated appendicitis. Via a typical inguinal herniorrhaphy incision, surgical exploration confirmed the diagnosis, and it was managed by opening the hernial sac to drain the abscess and reducing the appendix into the peritoneal cavity, followed by conventional tissue-based herniorrhaphy and a laparoscopic appendectomy. She was then discharged uneventfully and remained well for 11 months.

**Conclusions:**

Unlike the traditional definition of Amyand’s hernia, where the appendix is initially in the hernia sac, the current case demonstrated that Amyand’s hernia could be a type of delayed presentation following initial medical treatment of acute appendicitis. However, it can still be managed successfully by a conventional tissue-based herniorrhaphy followed by laparoscopic appendectomy.

## Background

Amyand’s hernia (AH) is defined as an inguinal hernia containing an inflamed or noninflamed appendix within the hernia sac [1]. AH can be seen in patients of all ages and consists of much less than 1% of all inguinal hernias [2, 3].

The symptoms and signs of AH with concomitant appendicitis generally present as nausea, vomiting and a nonreducible inguinal bulging mass with local tenderness and swelling. Typical signs of acute appendicitis, such as tenderness over McBurney’s point, psoas sign, and Rovsing sign, are absent in these patients due to the unique positioning of appendicitis [4, 5].

Although the exact mechanism of AH with concomitant appendicitis is not well clarified, several common hypotheses have been reported in the literature, including adhesion between the appendix and the inguinal sac followed by venostasis and hypoperfusion of appendix due to contraction of abdominal wall muscle [6, 7]; incarceration of the appendix leading to inflammation and swelling, which turns AH into a nonreducible hernia [8]. All the hypotheses of AH with simultaneous appendicitis have one thing in common: the preexistence of an inflamed appendix within the inguinal sac. Herein, we report one case of incarcerated AH which is caused by migration of a ruptured appendicitis 6 weeks after conservative treatment.

## Case presentation

**Fig. 1:**
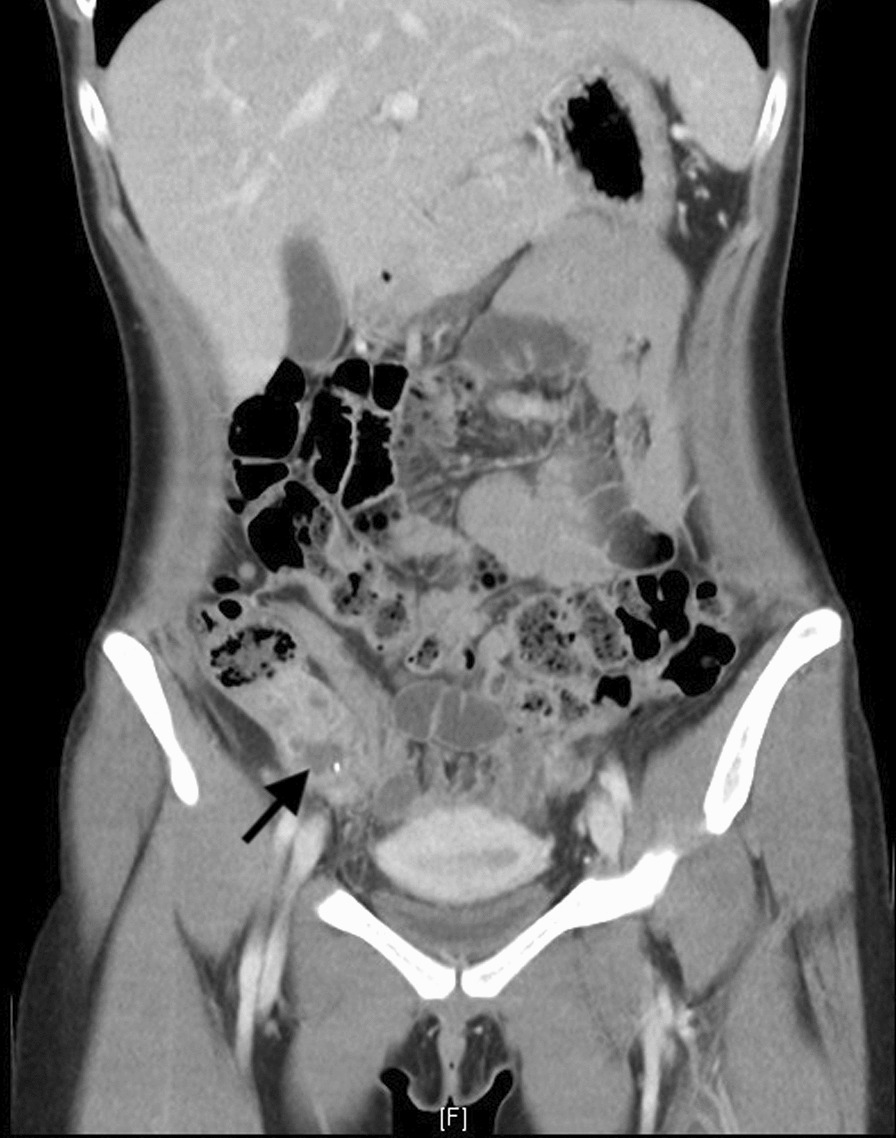
Initial contrast-enhanced abdominal CT revealed an engorged appendix with wall thickening, appendicolith and surrounding tumor formation, compatible with ruptured appendicitis with local abscess formation. (Arrow: Appendicolith with local abscess formation)

A 25-year-old healthy Taiwanese woman without any underlying medical disease or inguinal hernia history had experienced persistent right lower abdominal pain for 1 week. The pain was dull, progressive, and not related to food intake. Associated symptoms included anorexia and nausea. She was brought to the emergency department (ED) due to progressive symptoms, including a positive McBurney’s point tenderness, mild muscle guarding, mild leukocytosis with a left shift, and elevated CRP levels. Abdominal computed tomography (CT) showed an engorged appendix with wall thickening, appendicolith along with small amount of abscess (Fig. 1). No inguinal hernia was noted by CT, and the appendix was located in the paracecal position. Percutaneous drainage was contraindicated because of the engulfing surrounding intestinal loops and the more minimal volume of abscess. Therefore, she was treated with empiric antibiotics for a week and was discharged uneventfully.

Throughout the course of hospitalization, she did not complain of a bulging inguinal mass or inguinal pain. An interval appendectomy was scheduled at about three months after discharge.

**Fig. 2:**
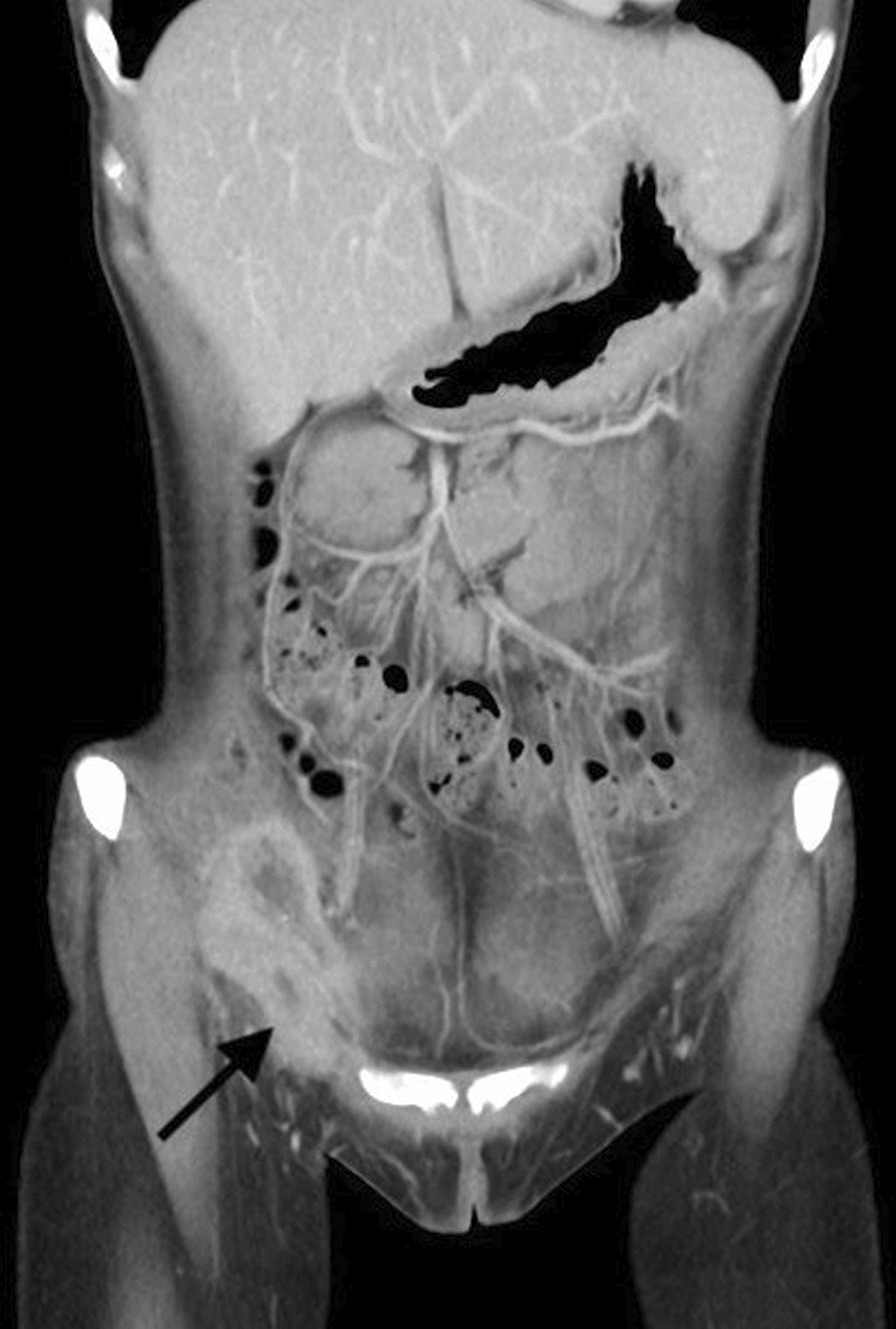
The follow-up contrast-enhanced abdominal CT revealed interval progression of the right lower abdominal abscess with transcompartment involvement, including the preperitoneal region and right inguinal canal, highly suspicious of incarcerated AH, secondary to ruptured appendicitis. (Arrow: Transcompartment inflammation into right inguinal canal)

**Fig. 3:**
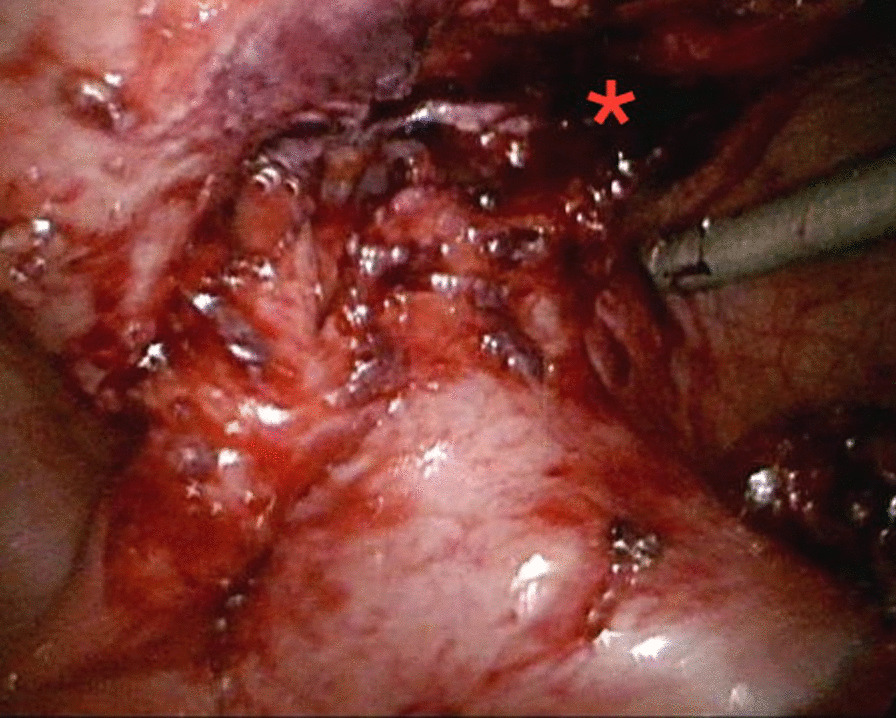
After detachment of the appendix from the inguinal canal, laparoscopic inspection clearly showed the internal ring of the right inguinal hernia (asterisk)

However, she came to the ED a month later due to a persistent painful right inguinal bulging mass for one week. It was firm, tender and nonreducible. No recent history of coughing or constipation was mentioned. Pelvic CT was then arranged and revealed an inflamed appendix with abscess formation in an indirect inguinal hernia raising the question of an Amyand’s hernia with a perforated appendicitis. (Fig. 2), including the preperitoneal region and right inguinal canal. It was most likely due to a perforated appendix incarcerated in the hernia sac. After thorough irrigation and debridement of the infected right inguinal region, right inguinal herniorrhaphy with McVay repair was performed by opening the sac, reducing the inflamed appendix into abdominal cavity, carefully avoiding contamination of the surgical field at inguinal region, and conducting high ligation of the sac. Use of a mesh-based repair was contraindicated because of the associated abscess. The reasons not to perform appendectomy in situ was that the cecal-appendiceal junction was still inside the peritoneal cavity and the appendiceal stump could not be safely secured if appendectomy was to be performed through the narrow opening of the sac. Furthermore, the peritoneal cavity had to be irrigated and cleared anyway. Therefore, closing the inguinal wound followed by laparoscopic appendectomy seemed to be the best choice under those circumstances. A laparoscopic appendectomy as well as irrigation of intra-abdominal abscess was then performed successfully (Fig. 3), with one Jackson Pratt drain left at the cul-de sac. The drain was successfully removed 5 days after the operation. (Fig. 3). The postoperative course was smooth without complications and the patient was discharged 5 days after the operation.

## Discussion and conclusions

AH is an uncommon but complicated type of inguinal hernia, arising in much less than 1% of all inguinal hernia cases [2]. It is an appendix inside the hernia sac (usually not inflamed). However, the trapped appendix in the hernia sac can become inflamed and the incidence of appendicitis within the inguinal hernia sac is reported to range from 0.07% to 0.13% of all inguinal hernias [9–11].

We herein reported an atypical type of AH that the inflamed appendix was initially located in the abdominal cavity and there was no history or physical findings of an inguinal bulge or an inguinal hernia. However, following conservative antibiotic-treated treatment for the perforated appendicitis, the appendix then migrated and trapped in a previous undiagnosed inguinal hernia. The likely pathophysiology might be that the local inflammation and surrounding intraperitoneal abscess has led to subsequent adhesions between the inflamed appendix and peritoneum. The peritoneum then became part of the hernia sac with the appendix trapped in it. When the appendicitis deteriorated, the incarcerated AH then became symptomatic.

A noninflamed AH can be treated with inguinal incision followed by inguinal herniorrhaphy, and the appendix is reduced into abdominal cavity with or without subsequent appendectomy [3, 12]. It is believed that such approach may keep the herniorrhaphy to be a clean surgery rather than a clean-contaminated surgery [2]. Although our case is a secondary Amyand’s hernia that was noted following initially conservative treatment of a perforated appendicitis, we took a similar approach to avoid extensive contamination of the operative field for herniorrhaphy. For our case, an additional advantage that appendectomy is performed after herniorrhaphy but not simultaneously with herniorrhaphy is that it is easier to clear intra-abdominal abscess via this approach. Furthermore, we chose laparoscopic appendectomy rather than open appendectomy for this patient because laparoscopic appendectomy is no longer a contraindication for perforated appendicitis at modern era [13].

In conclusion, our patient had a variant of AH that has never been reported before. The likely pathophysiology of this secondary AH was inflammation and adhesion of appendix to the part of peritoneum that subsequently became part of the indirect inguinal hernia sac. Herniorrhaphy followed by laparoscopic appendectomy provided good outcome for this patient.

## Data Availability

Data sharing is not applicable to this article as no datasets were generated or analysed during the current study.

## Abbreviations

AH: Amyand’s hernia
CT: Computed tomography
ED: Emergency department
